# Medication Adherence to Antidiabetic Therapy Among Patients With Tuberculosis and Diabetes in Ratlam District, Madhya Pradesh, India: A Facility-Based, Descriptive Cross-Sectional Study

**DOI:** 10.7759/cureus.106459

**Published:** 2026-04-05

**Authors:** Nitish Adawadkar, Dhruvendra Pandey, Umesh Sinha

**Affiliations:** 1 Department of Community Medicine, Dr. Laxmi Narayan Pandey Government Medical College, Ratlam, IND

**Keywords:** antidiabetic therapy, diabetes mellitus, india, medication adherence, tuberculosis

## Abstract

Background and objective

Tuberculosis (TB) and diabetes mellitus frequently co-occur in India and complicate the management of both conditions. Nevertheless, there is limited evidence on adherence to antidiabetic therapy among adults receiving TB care at district-level public health facilities. This study aimed to assess the levels of medication adherence and identify patient-reported barriers among adults with TB and diabetes attending two TB facilities in Ratlam district, Madhya Pradesh, India.

Methods

The facility-based cross-sectional descriptive study was derived from a parent research project conducted between January 2023 and December 2023 at the District Tuberculosis Centre and the TB Clinic of Government Medical College, Ratlam. Consenting adults aged over 18 years with newly diagnosed TB and coexisting diabetes were eligible. A pretested, structured questionnaire containing adherence and self-management items was used to collect data, and the current manuscript presents descriptive statistics.

Results

Out of the 135 respondents, 88 (65.2%) were men. The largest age group was 61 to 70 years (41.5%). Adherence to antidiabetic therapy was found to be low (80 participants, 59.3%), moderate (35 participants, 25.9%), and high (20 participants, 14.8%). Financial constraints (36.1%), medication side effects (26.2%), and the complexity of treatment regimens (18.3%) were the most commonly reported barriers to adherence. The majority of the participants received antidiabetic medicines in government institutions (78.5%).

Conclusions

The lack of adherence to antidiabetic therapy was widespread among adults with TB and diabetes in this setting. The most common barriers reported were financial barriers, adverse effects, and regimen complexity. These results support the need for improved adherence measurement and practical assistance in combined TB-diabetes care, and larger analytical studies are needed to identify independent determinants of nonadherence.

## Introduction

Diabetes mellitus is a significant public health concern at both national and global levels, and its incidence is increasing [1,2]. It has important implications for the management of tuberculosis (TB) since diabetes is a risk factor for active TB [3] and is associated with poorer TB treatment outcomes, such as treatment failure, death, and relapse [4]. In India, where communicable and noncommunicable conditions are increasingly co-occurring, TB-diabetes comorbidity has become a common challenge for district health services, rather than a rare clinical overlap [5,6].

In patients with both diseases, clinical treatment is more complex than managing either condition alone. Patients with comorbid diabetes may require management with both antitubercular therapy and antidiabetic medication, along with dietary management, glycemic control, and frequent clinic visits; they also experience a variable symptom burden during TB treatment. Adherence to antidiabetic treatment is particularly important in this setting, since inconsistent adherence to medications may worsen glycemic control and further complicate care in patients already at increased risk of adverse events. Several interacting factors affect adherence, including the cost of medications, access to care, adverse drug effects, regimen complexity, and the burden of frequent follow-up [7].

Even though medication adherence in type 2 diabetes has been investigated in both community- and hospital-based settings in India, the reported adherence levels vary across populations and according to the measurement tools used [8-10]. More to the point, most existing studies focus on individuals with diabetes alone rather than adults simultaneously receiving TB care. There is a lack of evidence regarding the use of district-level public-sector TB facilities, even though such facilities often serve patients who are older, from rural areas, financially vulnerable, and managing competing treatment demands. Such settings are also important at the programmatic level, as they represent routine service conditions where barriers to continuity of care may directly affect treatment delivery.

Describing adherence patterns and patient-reported barriers in this subgroup is therefore important not only for individual clinical management but also for planning integrated TB-diabetes care within routine public health services. The present study aimed to assess adherence to antidiabetic therapy and identify patient-reported barriers among adults with tuberculosis and diabetes attending two TB services in Ratlam district, Madhya Pradesh, India.

## Materials and methods

Study design and setting

This study is a facility-based descriptive cross-sectional analysis of adherence-related data collected from a larger parent research project conducted from January 2023 to December 2023 at the District Tuberculosis Centre, Ratlam, and the TB Clinic of Government Medical College, Ratlam, Madhya Pradesh, India. The parent project enrolled adults with newly diagnosed tuberculosis; the current report focuses specifically on adherence data from participants with coexisting diabetes.

Ethical approval

The study was approved by the Institutional Ethics Committee, Government Autonomous Medical College, Ratlam (approval no. GMC/RATLAM/2022/IEC/Approval/15; dated 29/08/2022). Written informed consent was obtained from all participants before data collection. The present manuscript presents a medication-adherence analysis derived from the ethically approved parent research project on diabetes mellitus and treatment outcomes in adults with newly diagnosed tuberculosis.

Participants

Adults aged 18 years or older with newly diagnosed tuberculosis and coexisting diabetes who attended either participating facility during the study period were eligible for inclusion. Newly diagnosed tuberculosis was defined according to the standard programmatic definition of a new TB case, that is, a patient who had never received anti-tubercular treatment or had received anti-TB drugs for less than one month. Eligible patients who provided written informed consent were included, while those who declined participation were excluded. As this manuscript presents a descriptive analysis of all eligible participants from the parent-project dataset during the recruitment period, a separate sample-size calculation for the present analysis was not performed. The final analytic sample included 135 participants.

Data collection and study variables

Data were collected using a pretested, structured questionnaire administered during the study visit. The questionnaire was pretested before formal data collection to improve clarity, sequencing, and local comprehensibility. It included sociodemographic and clinical items, treatment-related information, adherence-related items, and participant-reported barriers to medication adherence. The adherence section captured current antidiabetic therapy use, medication-taking regularity, missed or interrupted doses, source of medicines, and self-reported barriers, including financial difficulty, adverse effects, regimen complexity, perceived lack of benefit leading to use of traditional remedies, and difficulty accessing medication. For this manuscript, the main variables of interest were age, sex, education, residence, religion, body mass index, smoking status, type of antidiabetic therapy, source of antidiabetic medicines, adherence level, and reported barriers to adherence.

Blood glucose values recorded at the study visit included glucometer-based fasting and random blood sugar measurements. Within the parent study dataset, fasting blood sugar ≥126 mg/dL and random blood sugar ≥200 mg/dL were used as operational criteria for diabetes classification, in accordance with standard diagnostic thresholds [11]. Monotherapy was defined as the use of one antidiabetic drug, and combination therapy as the use of more than one antidiabetic drug at the time of assessment. For this manuscript, adherence categories were reported exactly as recorded in the original dataset without modification. For clarity, poor adherence was interpreted as low consistency of antidiabetic medication use with frequent missed or interrupted doses, moderate adherence as partial or intermittent regularity of medication use, and high adherence as largely regular medication use with minimal or no missed doses. These categories were treated as descriptive ordinal adherence levels, and no post hoc rescoring of the study instrument was undertaken.

Statistical analysis

Completed questionnaires were reviewed for completeness, coded, entered into Microsoft Excel 2019, and analyzed using IBM SPSS Statistics (IBM Corp., Armonk, NY). Because the objective of this manuscript was to describe adherence levels and summarize participant-reported barriers within the study setting, the analysis was conducted as a descriptive analysis. Continuous variables were summarized using mean and standard deviation (SD) where appropriate, while categorical variables were summarized as frequencies and percentages. No inferential or multivariable analyses were performed. Accordingly, the reported barriers should be interpreted as descriptive patient-reported reasons rather than independent determinants of nonadherence.

## Results

A total of 135 adults with TB and diabetes were included in this analysis. Men accounted for 88 (65.2%) of the participants, while women accounted for 47 (34.8%). The largest age group was 61-70 years (41.5%), followed by 51-60 years (21.5%) and 41-50 years (18.5%). Slightly more than half of the participants resided in rural areas (51.9%). Additional sociodemographic characteristics are presented in Table 1.

**Table 1:** Sociodemographic characteristics of the study participants (N = 135)

Variable	Category	N	%
Gender	Male	88	65.2
	Female	47	34.8
Age group, years	18-40	12	8.9
	41-50	25	18.5
	51-60	29	21.5
	61-70	56	41.5
	>70	13	9.6
Education	Postgraduate	1	0.7
	Graduate	34	25.2
	Diploma	68	50.4
	Middle school	2	1.5
	Primary school	4	3
	High school	25	18.5
	Illiterate	1	0.7
Residence	Rural	70	51.9
	Urban	65	48.1
Religion	Hindu	116	85.9
	Muslim	15	11.1
	Others	4	3

Most of the participants were overweight (73.3%), and 19.3% were classified as obese. More than half of the respondents were non-smokers (58.5%). Most of the participants used a single antidiabetic medication (71.1%), whereas 28.9% used multiple medications. Antidiabetic medications were obtained from the public setting by 78.5% of participants, and metformin was the most frequently prescribed drug (92.1%) (Table 2).

**Table 2:** Clinical and treatment-related characteristics Metformin was the most frequently prescribed antidiabetic medication (92.1%) BMI: body mass index

Variable	Category	N	%
BMI	Normal	10	7.4
	Overweight	99	73.3
	Obese	26	19.3
Smoking status	Yes	46	34.1
	No	79	58.5
	Not reported	10	7.4
Type of antidiabetic therapy	Monotherapy (1 drug)	96	71.1
	Combination therapy (>1 drug)	39	28.9
Medication source	Public setting	106	78.5
	Private setting	29	21.5

Poor adherence to antidiabetic therapy was observed in 80 participants (59.3%), moderate adherence in 35 (25.9%), and high adherence in 20 (14.8%) (Table 3). Financial constraints (36.1%), adverse effects of medication (26.2%), and the complexity of multiple drug regimens (18.3%) were the most common barriers to adherence (Table 4). Since the study was descriptive, these barriers were not interpreted as independent statistical predictors but as self-reported reasons.

**Table 3:** Distribution of medication adherence levels among study participants Distribution of study participants according to their level of adherence to antidiabetic therapy. Poor adherence indicates low consistency of medication use with frequent missed or interrupted doses; moderate adherence indicates partial or intermittent medication use; high adherence indicates largely regular medication use with minimal or no missed doses

Adherence category	N	%
Poor	80	59.3
Moderate	35	25.9
High	20	14.8

**Table 4:** Self-reported barriers to adherence among participants Descriptive summary of patient-reported barriers contributing to medication nonadherence

Reported barrier	N	% of reported reasons
Financial constraints	49	36.1
Adverse effects of medication	35	26.2
Complexity of multiple drug regimens	25	18.3
Perceived lack of benefit leading to self-medication with traditional remedies	13	9.8
Difficulty accessing medication	13	9.6

## Discussion

This study found that inadequate adherence to antidiabetic treatment was prevalent among adults with TB and diabetes receiving care at two TB facilities in Ratlam, with almost three out of five patients showing poor adherence and fewer than one out of six showing high adherence. This observation suggests that it may be challenging to achieve glycemic control concurrently in patients who are undergoing TB treatment, experiencing symptom burden, and having recurrent interactions with health facilities. This treatment instability is particularly relevant in tuberculosis-diabetes comorbidity, as these patients are already reported to be at higher risk of poor outcomes, and poor adherence to antidiabetic treatment can reasonably contribute to the complexity of care, as highlighted in recent literature describing the bidirectional relationship between tuberculosis and diabetes and the increased clinical burden when both diseases coexist [12], although this downstream effect was not directly investigated in the current analysis [4].

Another notable descriptive finding was the predominance of overweight and obesity in the cohort. Although TB is often associated with weight loss and undernutrition [4], this pattern is plausible because the study specifically included adults with coexisting diabetes, among whom overweight and obesity are common, particularly in type 2 diabetes [2]. In addition, body mass index was measured at a single study visit, and pre-illness body weight was not available; therefore, some participants may have presented after losing weight from a higher baseline before diagnosis or treatment.

The observed adherence pattern is largely congruent with the heterogeneity of the reported Indian studies of adults with diabetes, but direct comparisons should be made with caution, since study populations, care settings, and adherence measures vary. Sankar et al. reported that only 74% of diabetic adults in rural Kerala adhered to treatment [8], whereas Mukherjee et al. reported noncompliance in 42.3% of patients attending a diabetes clinic in Kolkata [9]. Arulmozhi and Mahalakshmy reported comparatively improved medication adherence in a hospital-based cohort in Puducherry [10].

Collectively, these studies indicate that estimates of adherence in India differ significantly across settings and are probably affected by the nature of the patient population and the environment in which care is provided. The reduced adherence in Ratlam, as compared to some diabetes-only clinic studies, could be because the current sample was recruited through TB services rather than routine noncommunicable disease clinics. Patients undergoing TB treatment are also prone to concurrent illness, increased clinic visits, competing priorities, and a higher overall treatment burden, all of which can decrease the consistency of antidiabetic medication adherence during tuberculosis treatment. Recent microbiological and epidemiological evidence has also emphasized that tuberculosis-diabetes comorbidity is associated with complex treatment pathways and greater clinical management challenges, which may further complicate adherence behaviour among affected patients [13].

The most common barrier to adherence was found to be financial constraints. The significance of this finding is that the cost of ensuring adherence in tuberculosis-diabetes treatment can extend beyond the direct price of drugs. Even when medicines are provided through public services, patients may still face costs related to transportation, loss of wages due to frequent visits, periodic out-of-pocket purchases, glucose monitoring, and the practical burden of adhering to recommended dietary requirements. The second most common barrier was adverse effects, which is understandable in a population exposed to multiple medications and experiencing overlapping symptoms. Regimen complexity was also commonly reported.

It is worth noting that, despite most participants being documented as receiving antidiabetic monotherapy, complexity was reported as a major obstacle. This suggests that patients may perceive complexity in the cumulative burden of managing both tuberculosis and diabetes, rather than solely in the number of antidiabetic medications. Previous public health analyses in India have similarly reported that integrated tuberculosis-diabetes care often introduces additional clinical and logistical challenges for patients, which may negatively influence medication adherence [14]. Such interpretation is consistent with general adherence literature and reports of operational difficulties in integrated tuberculosis-diabetes care in India [5-7].

The barriers identified in this study are not unique to tuberculosis and should not be interpreted as tuberculosis-specific determinants in a causal sense. Financial difficulty, adverse effects, regimen complexity, perceived lack of benefit, and difficulty accessing medication are well-recognized challenges in chronic disease management more broadly [7,15]. However, in the context of TB-diabetes comorbidity, tuberculosis may plausibly intensify these barriers by increasing treatment burden, clinic visits, symptom overlap, medication complexity, and difficulty maintaining glycemic control during active illness [5,6]. Because the present study was descriptive and did not include a comparison group of patients with diabetes without tuberculosis, the findings are best interpreted as general adherence challenges observed within a TB-diabetes population rather than evidence that tuberculosis alone caused poor adherence.

Programmatic implications of the current findings are also evident. Routine tuberculosis services are traditionally focused on microbiological follow-up and adherence to antitubercular therapy, whereas adherence to antidiabetic treatment may be less explicitly addressed within the same care pathway. These findings suggest that adherence to antidiabetic therapy should be more explicitly incorporated into routine tuberculosis care. Practical steps may include brief adherence assessments during follow-up visits, early evaluation of perceived adverse effects, medication reconciliation, and clear patient education on the importance of continuing antidiabetic therapy during tuberculosis treatment.

For patients reporting financial difficulty, ensuring reliable drug supply, strengthening linkages with local public noncommunicable disease services, and reducing unnecessary repeat visits may be more effective than generic advice alone. These implications align with the World Health Organization collaborative framework and Indian stakeholder analyses that call for improved coordination and more realistic support within tuberculosis-diabetes care pathways [5,6]. In addition, broader diabetes literature consistently identifies medication adherence as a key determinant of treatment outcomes and a persistent public health challenge in chronic disease management worldwide [15].

These findings should, however, be interpreted in light of the study limitations. The reported barriers reflect participant experiences but do not establish which factors are independently associated with poor adherence. Moreover, adherence was measured using questionnaire-based categories rather than objective measures of medication use, and the results may therefore be affected by recall and self-report bias. As a descriptive analysis limited to two public tuberculosis facilities in a single district, the findings cannot be generalized to all tuberculosis-diabetes care settings.

The current research also cannot determine whether nonadherence was temporary or persistent, or whether it was directly associated with glycemic indices or tuberculosis treatment outcomes. Future multicenter studies are recommended to use well-validated adherence measures, incorporate variables such as diabetes duration, glycated hemoglobin, stage of tuberculosis treatment, pill burden, and socioeconomic status, and apply multivariate analyses to distinguish modifiable barriers from underlying patient characteristics.

Limitations

This study has several limitations. First, adherence was measured using a questionnaire-based method and is therefore prone to recall and social desirability bias. Second, there was no objective measure of adherence, such as pill counts, pharmacy refill data, or electronic monitoring, to support self-report. Third, as a descriptive study, no inferential or multivariate analyses were conducted; thus, the reported barriers cannot be considered independent predictors. In addition, the study did not include a comparison group of patients with diabetes without tuberculosis, making it difficult to determine whether the observed barriers were specific to tuberculosis or reflected broader challenges in chronic disease management. Fourth, the analysis did not include important covariates such as duration of diabetes, HbA1c, phase and severity of tuberculosis treatment, comorbidities not descriptively captured, and socioeconomic status. Lastly, the study was conducted in two facilities within a single district, which limits the generalizability of the findings to other program settings.

## Conclusions

Poor adherence to antidiabetic therapy was common among adults with tuberculosis and diabetes in this setting. Financial constraints, adverse effects, and regimen complexity were the most commonly reported barriers. These results indicate that antidiabetic adherence needs to be more carefully evaluated within TB services, rather than being considered a secondary concern when TB patients are receiving care. The barriers identified in this study are not unique to tuberculosis, but they may be amplified in the setting of TB-diabetes comorbidity because of the added burden of managing two conditions simultaneously. The identified barriers may be addressed through integrated counseling, early management of medication side effects, and improved access to antidiabetic therapy. To identify independent predictors of nonadherence, as well as to clarify the effect of adherence on clinical outcomes in TB-diabetes comorbidity, larger multicenter analytical studies are still required.
